# Why are psychiatric imaging methods clinically unreliable? Conclusions and practical guidelines for authors, editors and reviewers

**DOI:** 10.1186/1744-9081-8-46

**Published:** 2012-09-01

**Authors:** Stefan Borgwardt, Joaquim Radua, Andrea Mechelli, Paolo Fusar-Poli

**Affiliations:** 1 Department of Psychiatry, University of Basel, Petersgraben 4, Basel, CH 4031, Switzerland; 2 Department of Psychosis Studies, Institute of Psychiatry, King’s College London, De Crespigny Park, London, SE5 8AF, UK

**Keywords:** Guidelines, Neuroimaging, Magnetic resonance imaging, Region-of-interest, Voxel-based morphometry, Meta-analyses

## Abstract

No reliable anatomical or functional alterations have been confirmed in psychiatric neuroimaging; however it can become reliable with translational impact on clinical practice when considering crucial methodological issues. We provide guidelines to authors, editors and reviewers in the implementation/evaluation of neuroimaging studies to bend neuroimaging to be more than basic neuroscience.

## Background

More than three decades after Johnstone’s first computerised axial tomography of the brain of individuals with schizophrenia [1], no consistent or reliable anatomical or functional alterations have been univocally associated with any mental disorder and no neurobiological alterations have been ultimately confirmed in psychiatric neuroimaging.

A number of methodological problems may underlie the inconsistencies across studies and the difficulty of identifying reliable results. Heterogeneity in psychiatric neuroimaging originates from multiple differences across studies: in conceptual issues underlying psychiatric diagnoses and psychopathology [2,3], the inclusion criteria for and the clinical characteristics of psychiatric samples [4]; the use of different paradigms and designs [4], and the use of different forms of image acquisition and image analysis [5].

## Discussion

The latter point is critically addressed by the recent study of Ioannidis [6]. He stated that *“the excess significance may be due to unpublished negative results, or it may be due to negative results having been turned into positive results through selective exploratory analyses”*[6]. Because of multiple comparisons across different brain regions, reporting of regions of interest (ROIs) can be guided by post-hoc significance of the results, with the whole brain results remaining unpublished [6]. Additionally, when there are many ROI analyses that can be performed, only one of them, the one with the best results, may be presented [6]. These practices limit the correct *localization* of the potential brain abnormalities, which should be based on a *whole-brain* analysis of the differences between patients and controls. To make an analogy, it’s as if an attorney decides to investigate only an arbitrary subgroup of the suspects of a crime, and not to report any proof, which may involve individuals which he wants to keep untarnished.

As Ioannidis acknowledged these concerns do mainly refer to morphometry studies and not directly extend to automated whole-brain voxel-based studies or functional imaging studies. In particular, voxel-based meta-analyses have the potential to overcome the limited sample size of individual studies revealing structural differences at specific brain coordinates rather than differences in volumes of pre-specified ROIs. A recently developed meta-analytic method, Signed Differential Mapping [7,8], considers null findings as well and thus attenuates the disproportionate influence of single study data sets. However, even meta-analyses of voxel-based studies are grounded on the available published results, which often do not report null findings. In this regard, it must be noted that no meta-analytic method can detect an abnormality if this is deliberately not reported in the individual studies, e.g. by repeating the analysis with different parameters until the finding disappears. This may be the case of abnormalities in regions not thought to be related to the disorder, which may be “felt” to be false positives or artifacts [8] by the authors of the studies and by the peer-reviewers.

## Conclusions and practical guidelines for authors, editors and reviewers

Only by overcoming these biases, the results of psychiatric neuroimaging can become more reliable and have a translational impact on clinical practice. The study by Ioannidis represents a milestone in psychiatric imaging, pointing to crucial methodological issues at the level of imaging analysis. Although the Ioannidis study makes general recommendations, this manuscript tries to formulate a checklist of practical guidelines for authors, editors and reviewers that are easy to implement and follow. This may help to ultimately bend psychiatric neuroimaging to be something more than basic neuroscience:

i. With an increasing number of ways of preprocessing the data becoming available, this should be described in enough detail by the authors to allow exact replication;

ii. ROI studies (employing preselected masks or adopting Small Volume Corrections) should first report standard whole brain results and acknowledge if no significant clusters were detected at whole brain level before presenting the ROI findings;

iii. Both ROIs and whole brain studies should first report the results significant at p < 0.05 corrected for multiple comparisons (i.e. FWE, FDR, Montecarlo) and then employ more liberal thresholds;

iv. When several ROIs are used, correction for multiple comparisons should be based on a mask which includes all of them rather than considering each ROI separately;

v. Authors should be encouraged to blind the statistical analyses of the imaging datasets to avoid ROI analyses be built post-hoc on the basis of the results;

vi. All studies should report a statistical analysis modelling an agreed set of possible confounding variables; these could include, for instance, gender, age and handedness. In addition, studies would have the option of reporting further statistical analyses modelling additional study-specific confounding variables;

vii. All studies should acknowledge the number of analyses or brain correlations performed, giving a clear rationale for each, to avoid conducting exploratory analyses and reporting the most significant result;

viii. The potential overlapping of the patient and control group with previously published studies should be clearly acknowledged, and the spatial coordinates always reported, to assist future voxel-based meta-analyses in the field;

ix. Peer-reviews should be as strict when assessing the methods of a study reporting abnormalities in expected brain regions, as when assessing the methods of a study not finding any expectable finding;

x. Acceptance or rejection of a manuscript should not depend on whether abnormalities are detected or not, nor on the specific brain regions found to be abnormal.

## Competing interests

The authors’ declare that they have no competing interests.

## Authors’ contributions

SB and PFP conceived these guidelines and drafted the manuscript. JR and AM helped discussing the limitations of imaging analysis. All authors read and approved the final manuscript.
